# Supplementary material for: Alterations in Circulating Monocytes Predict COVID-19 Severity and Include Chromatin Modifications Still Detectable Six Months after Recovery
Source: Biomedicines. 2021 Sep 17;9(9):1253. doi: 10.3390/biomedicines9091253 (PMC8471575; doi:10.3390/biomedicines9091253)
Supplement: Supplementary file 1 [file biomedicines-09-01253-s001.zip › biomedicines-1354639-supplementary.pdf]

SUPPLEMENTARY FIGURES

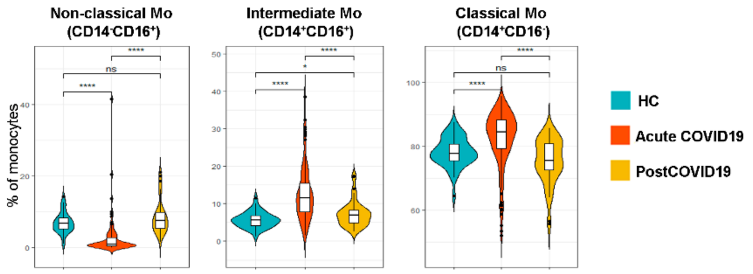

Supplementary figure S1. Comparison of monocyte subsets in HC, acute and post-COVID-19 patients. *, $p<0.05$; **, $p<0.01$; ***, $p<0.001$; ****, $p<0.0001$.

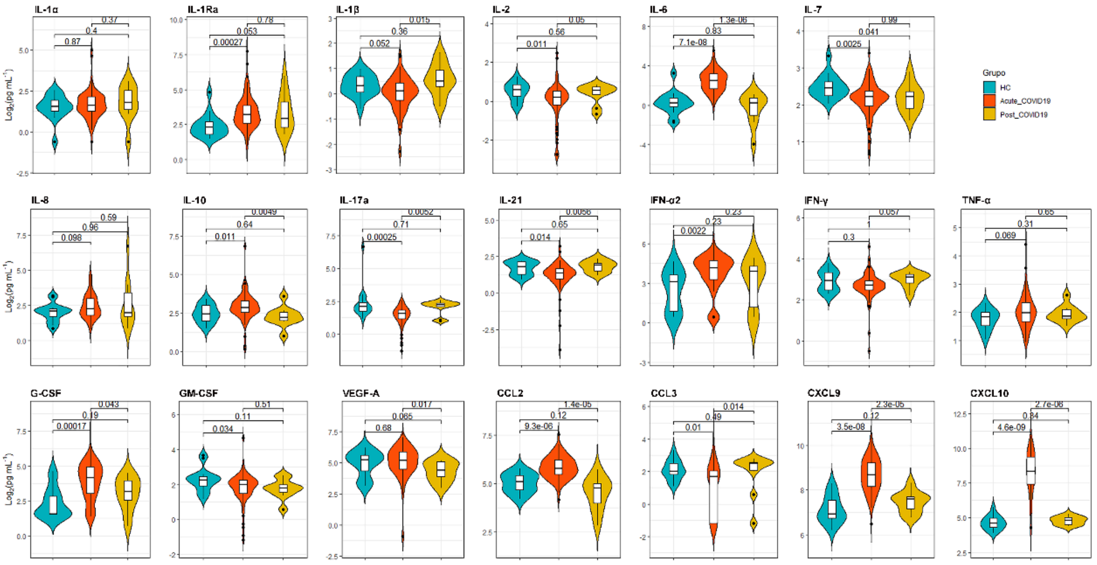

Supplementary figure S2. Plasma cytokine levels in HC, acute and post-COVID-19 patients.

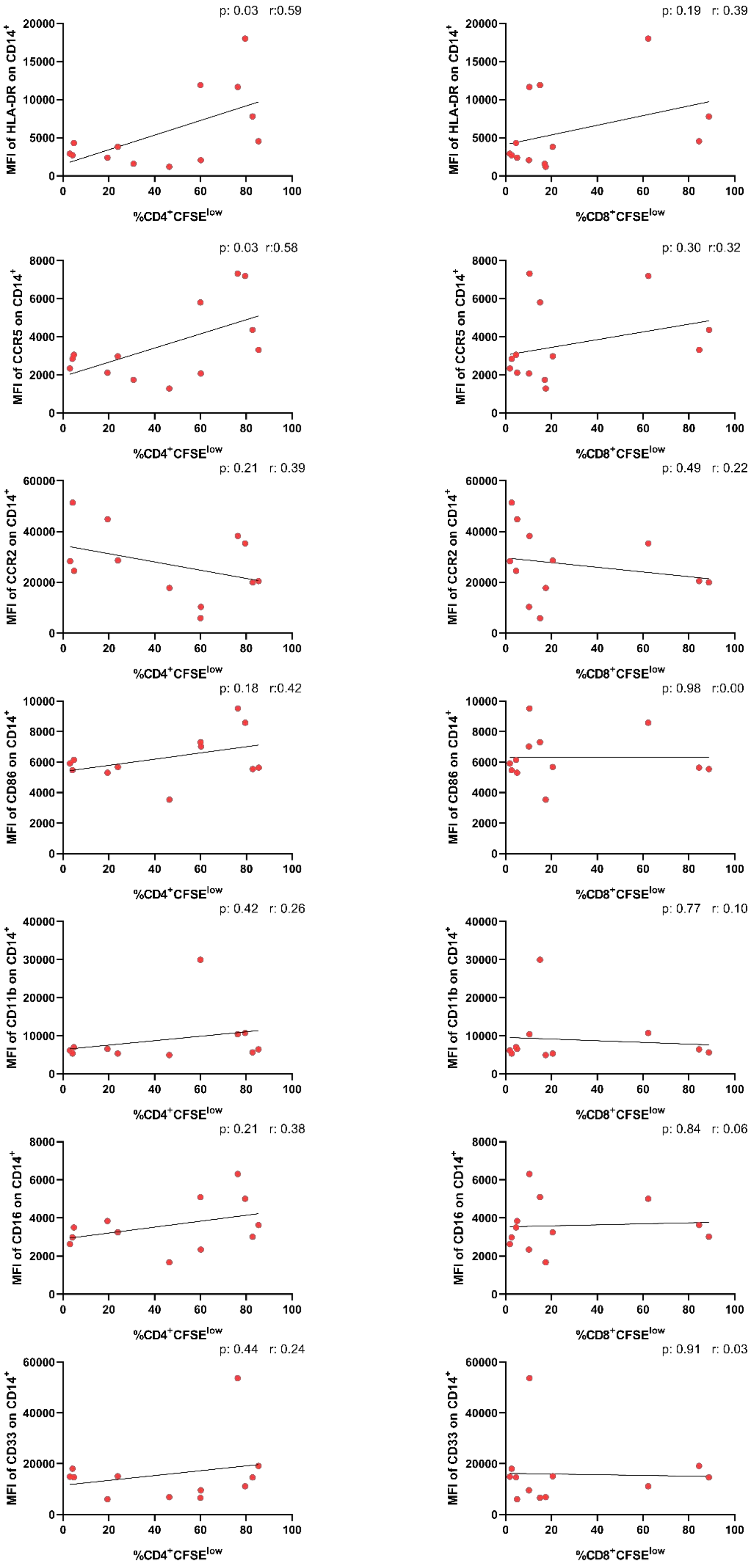
p: 0.03 r:0.59
MFI of HLA-DR on CD14$^+$
%CD4$^+$CFSE^{low}
p: 0.19 r: 0.39
MFI of HLA-DR on CD14$^+$
%CD8$^+$CFSE^{low}
p: 0.03 r:0.58
MFI of CCR5 on CD14$^+$
%CD4$^+$CFSE^{low}
p: 0.30 r:0.32
MFI of CCR5 on CD14$^+$
%CD8$^+$CFSE^{low}
p: 0.21 r: 0.39
MFI of CCR2 on CD14$^+$
%CD4$^+$CFSE^{low}
p: 0.49 r: 0.22
MFI of CCR2 on CD14$^+$
%CD8$^+$CFSE^{low}
p: 0.18 r:0.42
MFI of CD86 on CD14$^+$
%CD4$^+$CFSE^{low}
p: 0.98 r:0.00
MFI of CD86 on CD14$^+$
%CD8$^+$CFSE^{low}
p: 0.42 r: 0.26
MFI of CD11b on CD14$^+$
%CD4$^+$CFSE^{low}
p: 0.77 r: 0.10
MFI of CD11b on CD14$^+$
%CD8$^+$CFSE^{low}
p: 0.21 r: 0.38
MFI of CD16 on CD14$^+$
%CD4$^+$CFSE^{low}
p: 0.84 r: 0.06
MFI of CD16 on CD14$^+$
%CD8$^+$CFSE^{low}
p: 0.44 r: 0.24
MFI of CD33 on CD14$^+$
%CD4$^+$CFSE^{low}
p: 0.91 r: 0.03
MFI of CD33 on CD14$^+$
%CD8$^+$CFSE^{low}

Supplementary figure S3. Correlation between monocyte surface markers and CD4 and CD8 T cell proliferation in acute COVID-19 patients at ER arrival.

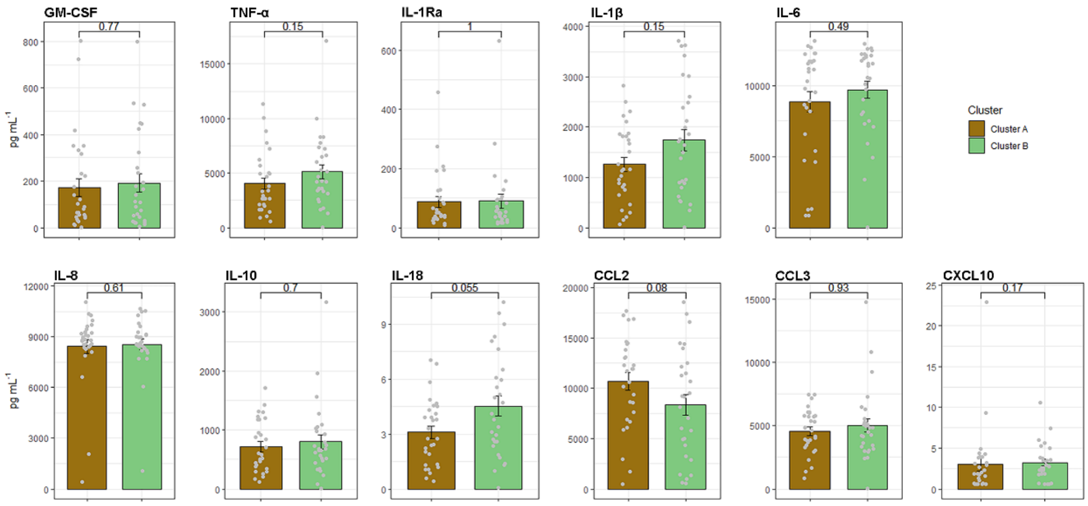

Supplementary Figure S4. Comparison of cytokine production by LPS-stimulated monocytes from cluster A and B.

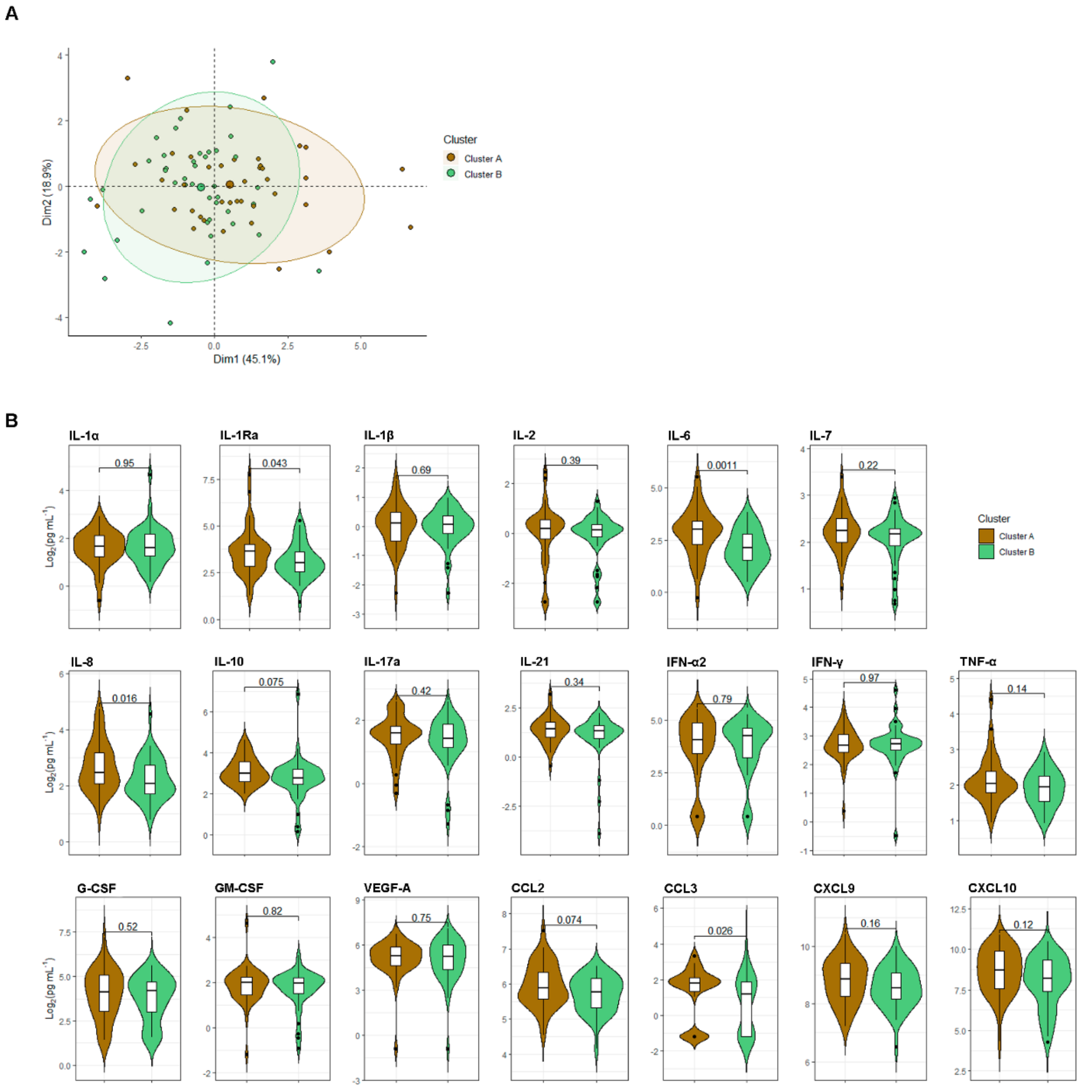

Supplementary Figure S5. Plasma cytokine levels in cluster A versus B acute COVID-19 patients. (A) Principal component analysis (PCA) and (B) individual cytokine levels in cluster A versus B. *, p<0.05; **, p<0.01; ***, p<0.001; ****, p<0.0001.

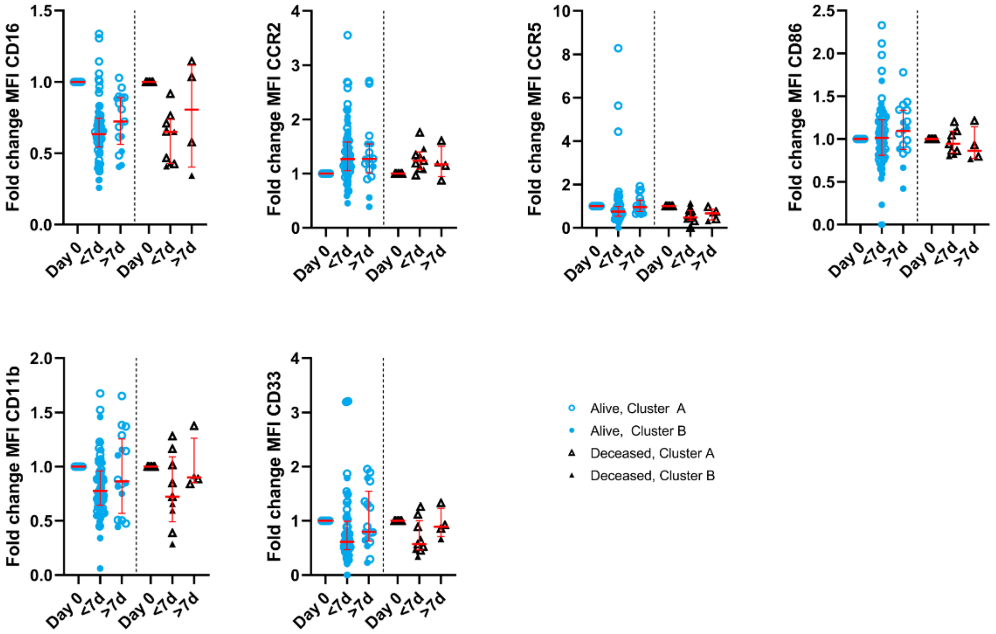

Supplementary Figure S6. Fold change of monocyte surface markers throughout hospitalization. Blue points represent discharged patients and black points patients who died. Alive (n=68); deceased (n=8).

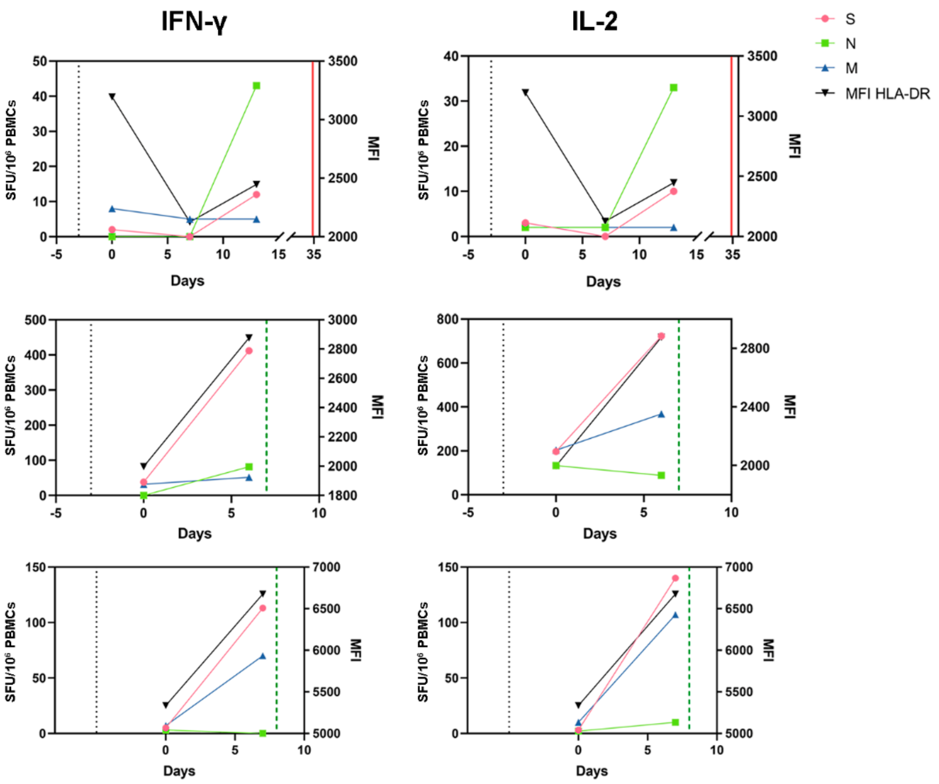

Supplementary Figure S7. Longitudinal analysis of HLA-DR expression on monocytes and specific T cell response against SARS-CoV-2: correlation in individual examples. X-axes represent total days (Days of symptoms: <0; days of hospitalization: >0). Day 0 represents emergency room arrival day. Vertical black, dashed lines represent the day of symptoms onset. Vertical red, solid lines represent the day of dying. Vertical green, dashed lines represent the day of discharging

SUPPLEMENTARY TABLES

Table S1. Characteristics of acute COVID-19 patients at emergency room arrival and treatment received

**Variables**	**Acute COVID19 (N=131)**	**Cluster A (N=60)**	**Cluster B (N=71)**	**p**
Age (Median, [IQR])	63 [51-77]	67 [52-81]	62 [50-71]	0.13
Sex, male (n, [%])	89 [67.9%]	43 [71.7%]	47 [66.2%]	0.46
Comorbidities (n, [%])				
Obesity	12 [9.2%]	5 [8.3%]	7 [9.7%]	1
Hypertension	59 [45%]	30 [50%]	29 [40.3%]	0.29
Diabetes mellitus	20 [15.3%]	7 [11.7%]	13 [18.1%]	0.34
Dyslipidemia	49 [37.4%]	19 [31.7%]	30 [41.7%]	0.28
ARDS classification, on test day (n, [%])				0.68
None	73 [55.7%]	31 [51.7%]	42 [59.2%]	
Mild	34 [25.9%]	16 [26.7%]	18 [25.4%]	
Moderate	9 [6.9%]	4 [6.6%]	5 [7.0%]	
Severe	15 [11.5%]	9 [15.0%]	6 [8.4%]	
Laboratory parameters (Median, [IQR])				
SpO_2/FiO_2	339 [267-438]	339 [269-438]	343 [300-443]	0.33
Platelets (10^3/µL)	219 [170-292]	211 [175-300]	225 [167-290]	0.66
Monocytes (10^3/µL)	0.5 [0.4-0.7]	0.5 [0.4-0.7]	0.5 [0.4-0.6]	0.74
Neutrophil/Lymphocyte ratio	7.2 [4.1-12]	8.8 [4.1-15]	6.5 [4.1-10]	0.12
LDH (U/L)	395 [322-480]	405 [325-546]	389 [307-459]	0.10
Albumin (g/dL)	3.9 [3.7-4.2]	3.8 [3.6-4.1]	4 [3.7-4.2]	0.09
C-reactive protein (mg/dL)	10.5 [5.8-17.0]	13.0 [7.6-24.4]	9 [4.8-14.6]	**0.007**
IL6 (pg/mL [a])	32.5 [19.6-78.9]	50.0 [26.8-116.5]	26.2 [14.4-52.0]	**0.0002**
ALT (U/L)	37 [20-64]	34 [19.5-56.5]	40.5 [21-72.8]	0.25
AST (U/L)	46 [31-69]	47 [29-61]	44 [33-71]	0.66
Fribrinogen (mg/dL)	790 [711-938]	784 [678-955]	791 [746-915]	0.90
D-dimer (ng/mL)	736 [503-1233]	745 [561-1126]	729 [483-1237]	0.48
Viral load, Ct (Median, [IQR])	28.2 [23.4-32.1]	25.3 [22.5-32.3]	28.9 [24.5-31.9]	0.43
Days from illness onset to hospital admission (Median, [IQR])	7 [4-10]	7 [4-9]	8 [5-10]	0.12
Days of hospital stay (Median, [IQR])	8 [6-13]	7 [5-12]	7 [5-11]	0.43
Treatment during hospital stay (n,[%])				
Remdesivir	45 [34.4%]	25 [41.7%]	20 [28.2%]	0.14
Corticosteroids	119 [90.8%]	56 [93.3%]	63 [88.7%]	0.55
Azithromycin	67 [51.1%]	31 [51.7%]	36 [50.7%]	1
Ceftriaxone	113 [86.3%]	52 [86.7%]	61 [85.9%]	1
Tocilizumab	31 [23.7%]	14 [23.3%]	17 [23.9%]	1
Oxygen therapy	112 [85.5%]	52 [86.7%]	60 [84.5%]	0.81
Mechanical ventilation	44 [33.6%]	24 [40.0%]	20 [28.2%]	0.19
Hiperimmune plasma	7 [5.3%]	4 [6.7%]	3 [4.2%]	0.70
Thromboprophylaxis	127 [96.9%]	58 [96.7%]	69 [97.2%]	1
ICU admission (n,[%])	21 [16.0%]	14 [23.3%]	7 [9.7%]	**0.05**
Exitus (n, [%])	18 [13.7%]	14 [23.3%]	4 [5.6%]	**0.03**

[a] Analyzed by Cytometry Bead Assay

Table S2. Characteristics of post-COVID-19 patients

**Variables**	**Post-COVID-19 (N=52)**
Age (Median, [IQR])	65 [50-74]
Sex, male (n, [%])	29 [55.8%]
Comorbidities (n, [%])	
Obesity	2 [3.8%]
Hypertension	19 [36.5%]
Diabetes mellitus	9 [17.3%]
Dyslipidemia	16 [30.8%]
Laboratory parameters at hospital admission (Median, [IQR])	
Oxigen saturation	98 [95-98]
Platelets (x10^3 cells/µL)	219 [182-241]
Monocytes (x10^3 cells/µL)	0.5 [0.3-0.7]
Neutrophil/Lymphocyte ratio	5.3 [3.8-8.4]
LDH (U/L)	366 [305-375]
Albumin (g/dL)	4.1 [3.8-4.3]
C-reactive protein (mg/dL)	9.2 [4.8-12.9]
Fribrinogen (mg/dL)	767 [675-835]
Current laboratory parameters (Median, [IQR])	
SpO_2/FiO_2	> 96%
Platelets (x10^3 cells/µL)	226.5 [197.0-263.8]
Monocytes (x10^3 cells/µL)	0.5 [0.4-0.6]
Neutrophil/Lymphocyte ratio	1.6 [1.4-2.0]
LDH (U/L)	192 [171-219]
Albumin (g/dL)	4.7 [4.5-4.8]
C-reactive protein (mg/dL)	0.14 [0.07-0.30]
Fribrinogen (mg/dL)	415 [358-457]
Long-COVID symptoms (n, [%])	
Dyspnea	8 [15.4%]
Asthenia	8 [15.4%]
Tachycardia	3 [5.8%]
Myalgia	6 [11.5%]
Arthralgias	6 [11.5%]
